# Efficacy of bednets with dual insecticide-treated netting (Interceptor® G2) on side and roof panels against *Anopheles arabiensis* in north-eastern Tanzania

**DOI:** 10.1186/s13071-022-05454-w

**Published:** 2022-09-15

**Authors:** Njelembo J. Mbewe, Mark W. Rowland, Janneke Snetselaar, Salum Azizi, Graham Small, Derric D. Nimmo, Franklin W. Mosha

**Affiliations:** 1 Department of Disease Control, London School of Hygiene and Tropical Medicine, London, UK; 2 Kilimanjaro Christian Medical University College, Pan African Malaria Vector Research Consortium, Moshi, Tanzania; 3 Innovative Vector Control Consortium, Liverpool School of Tropical Medicine, Liverpool, UK

**Keywords:** Insecticide resistance, Alphacypermethrin, Chlorfenapyr, *Anopheles arabiensis*, Partially treated nets, Long-lasting insecticidal nets

## Abstract

**Background:**

Optimising insecticide use and managing insecticide resistance are important to sustain gains against malaria using long-lasting insecticidal nets (LLINs). Restricting insecticides to where mosquitoes are most likely to make multiple contacts could reduce the quantity of insecticide needed to treat the nets. Previous studies have shown that nets partially treated with a pyrethroid insecticide had equivalent mortality compared to a fully treated net. This study compared the efficacy of: (i) whole Interceptor® G2 nets (IG2; a dual-active LLIN containing alpha-cypermethrin and chlorfenapyr), (ii) nets with roof panels made of IG2 netting, (iii) nets with side panels made of IG2 netting and (iv) whole untreated nets as test nets.

**Methods:**

The study was conducted in cow-baited experimental huts, Moshi Tanzania, using a four-arm Latin square design. Test nets had 30 holes cut in panels to simulate a typical net after 2–3 year use. The trial data were analysed using generalized linear models with mortality, blood-feeding, exophily and deterrence against wild free-flying *Anopheles arabiensis* as outcomes and test nets as predictors.

**Results:**

Mortality was significantly higher in the nets with roof IG2 [27%, *P* = 0.001, odds ratio (OR) = 51.0, 95% CI = 4.8–546.2), side IG2 (44%, *P* < 0.001, OR = 137.6, 95% CI = 12.2–1553.2] and whole IG2 (53%, *P* < 0.001, OR = 223.0, 95% CI = 19.07–2606.0) nettings than the untreated (1%) nets. Mortality was also significantly higher in the whole IG2 net compared to the net with roof IG2 netting (*P* = 0.009, OR = 4.4, 95% CI = 1.4–13.3). Blood feeding was 22% in untreated, 10% in roof IG2, 14% in side IG2 and 19% in whole IG2 nets. Exiting was 92% in untreated, 89% in roof IG2, 97% in side IG2 and 94% whole IG2 nets.

**Conclusion:**

The results show that although the roof-treated IG2 net induced greater mortality compared to untreated nets, its efficacy was reduced compared to whole IG2 nets. Therefore, there was no benefit to be gained from restricting dual-active ingredient IG2 netting to the roof of nets.

**Graphical Abstract:**

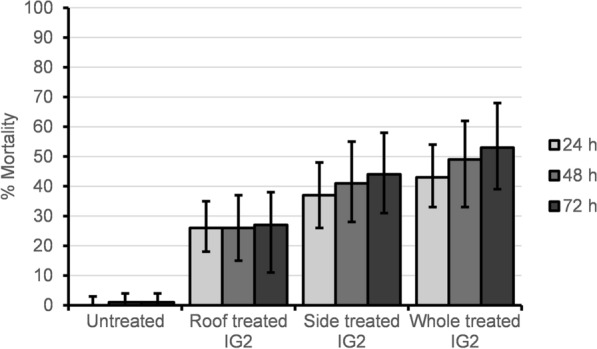

**Supplementary Information:**

The online version contains supplementary material available at 10.1186/s13071-022-05454-w.

## Background

Vector control using long-lasting insecticidal nets (LLINs) is the main malaria control strategy [1]. LLINs contributed approximately 68% of the decline in malaria cases between 2000 and 2015 [2]. During this period, the main active ingredients in LLINs were pyrethroids because of their high effectiveness for malaria vector control, their low human toxicity and low cost [1]. However, widespread resistance to pyrethroids has been reported from many countries and the problem is growing fast [3]. This reduces the impact of LLINs in the fight against malaria [4, 5]. As a result, there has been a drive to develop novel LLINs treated with dual-active ingredients to counter pyrethroid resistance [6–10]. These LLINs, collectively known as “new generation nets”, are treated with pyrethroids and other compounds such as the synergist, piperonyl butoxide or an insecticide from a class with a different mode of action from pyrethroids [1, 11, 12]. The new generation nets based on pyrethroid and PBO function by neutralising metabolic forms of pyrethroid resistance conferred by P450 mono-oxygenases [12]. Others simultaneously expose host-seeking vectors to two insecticides so that those not killed by the pyrethroid component are killed or sterilised by the other insecticide [1, 10, 12]. Some of the insecticides used in new generation LLINs with different modes of action to pyrethroids include an insect growth regulator, pyriproxyfen [12], and a cellular respiratory disruptor, chlorfenapyr [9, 10]. The continuing use of pyrethroids in new generation nets as one of the active ingredients is mainly due to its irritancy property, which enhances personal protection by preventing blood feeding [1].

Laboratory and semi-field study data suggest that the majority of *Anopheles gambiae* and *An. arabiensis* make multiple brief contacts on the roof compared with side panels of LLINs when seeking blood meals [4, 11, 13, 14]. This behaviour has been attributed to mosquito attraction to thermal convection of odour plumes of carbon dioxide that emanate from human hosts under bed nets [15]. It may also depend on indoor air movement and ambient temperature, with cool, still air conditions promoting activity of *An. gambiae* on the roof of an untreated net while warm, still air conditions reduce activity on the roof [16]. Additionally, cross-draughts can increase the activity of host-seeking *An. gambiae* on the sides of untreated nets [16]. The host-seeking behaviour of malaria vectors at the top of LLINs has sparked research interest [17] that has opened possibilities of strategically designing nets to optimise insecticide use while controlling pyrethroid resistant vectors. Restricting the application of insecticides to where malaria vectors make multiple contacts with the net could potentially reduce the amount of insecticide needed and the cost of treating nets. In addition, insecticides that may appear too expensive at first sight could compete with generic pyrethroid-only nets if less was required on a net.

Oxborough et al. [17] showed in experimental huts that roof, side and whole nets treated with lambda-cyhalothrin at 18 mg/m^2^ elicited similar levels of mortality (top only 39.2%, sides only 39.6%, all surfaces 39.7%) against *An. arabiensis* in northern Tanzania. They also showed significant reduction in blood feeding in the fully treated nets compared to roof-treated nets [17]. The first PBO synergist net to be developed, PermaNet® 3.0, had the PBO restricted to the roof of the net [18]. This was shortly followed by Olyset™ Plus with PBO impregnated on all panels of the net [19]. DawaPlus 3.0 also had PBO restricted to the roof, whereas DawaPlus 4.0 had PBO-treated roof and sides [20]. Despite this, the efficacy of roof, sides and whole new generation nets treated with a mixture of insecticides against resistant malaria vectors remains to be confirmed. Determining their efficacy and properties could lead to new strategies for rational insecticide use based on dual-active ingredient LLINs [17]. Interceptor® G2 (BASF, Ludwigshafen, Germany), IG2, is a new generation LLIN coated with a wash-tolerant mixture of insecticides alphacypermethrin and chlorfenapyr [9, 10, 12, 20].

Whereas alphacypermethrin is a fast-acting neurotoxic pyrethroid, the pyrrole chlorfenapyr is a more slowy acting pro-insecticide that, after metabolic activation, disrupts cellular respiration in the flight muscle of the mosquito and conversion of ADP to ATP and shows no cross resistance to insecticides that act on the nervous system of insects [10, 12]. Both alphacypermethrin and chlorfenapyr are adulticidal, so IG2 serves as a model candidate new generation LLIN for efficacy studies of insecticide treatment restricted to roof and side LLIN panels. Therefore, in the present study, the efficacy of nets with IG2 material on the roof panel only, those with IG2 material on the side panel only and whole IG2 LLIN was compared in terms of inducing mortality, blood feeding inhibition, exiting and deterrence in free-flying wild *An. arabiensis.*

## Methods

### Study site

The study was undertaken between June and September 2021 in Pasua (S03º22.764’; E37º20.793’) [21], lower Moshi Tanzania. The site is one of the Kilimanjaro Christian Medical University College- Pan African malaria vector research consortium (KCMUCo-PAMVERC) Insecticide Test Facility field sites. It has seven East African style experimental huts [22] adjacent to the Lower Moshi rice irrigation scheme [21]. There are two rice-planting periods: June–September and November﻿–January. *Anopheles arabiensis* is the major Anopheline species in the area [17, 21] and tends to peak in density during the rice planting seasons. *Anopheles arabiensis* in Lower Moshi exhibits moderate pyrethroid resistance driven by overexpression of P450 mixed function oxidases [23, 24] and zoophilic feeding behaviour [25, 26].

### Preparation of mosquito net treatments

**Fig. 1:**
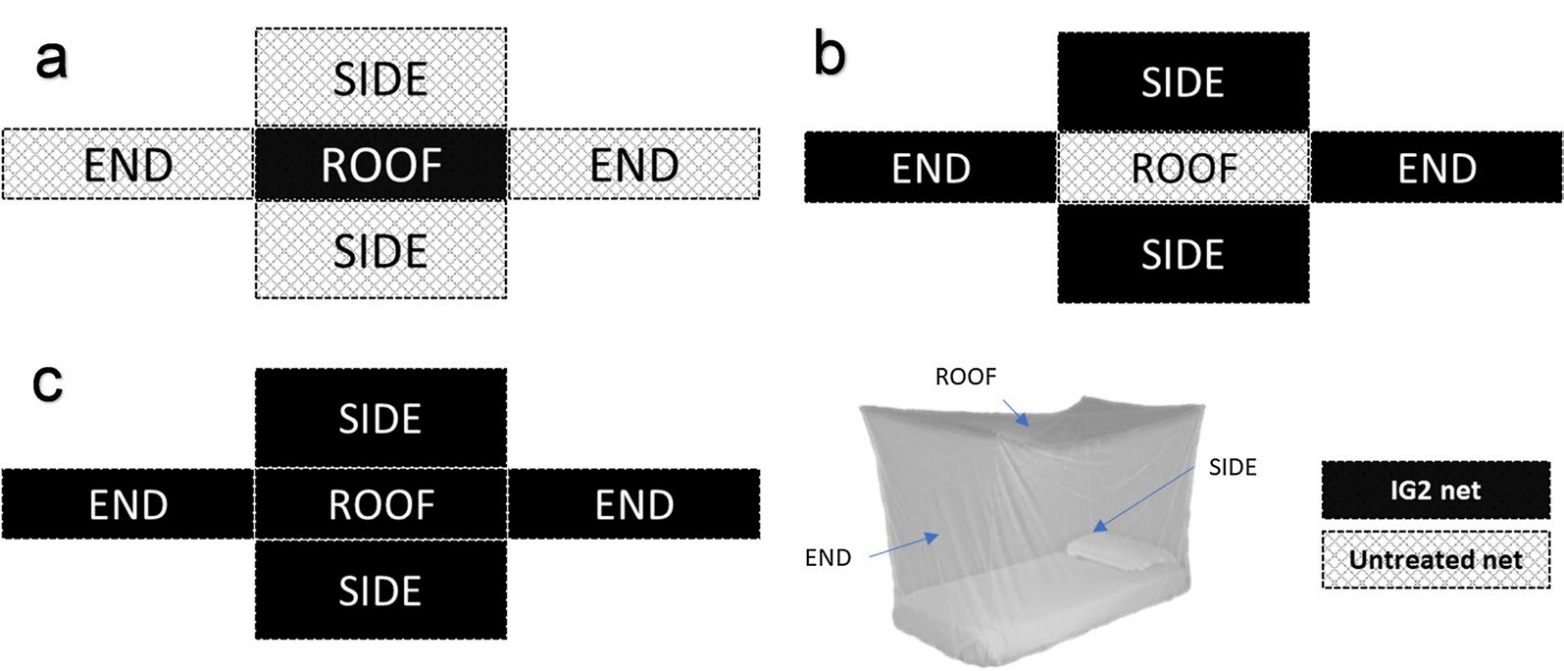
Schematic diagram of different LLIN designs used in trial arms; roof-treated IG2 (**A**), side-treated IG2 (**B**) and IG2 (**C**) nets. Black represents IG2 net panels and light grey represents untreated net panels

Preparation of the nets was performed at the KCMUCo-PAMVERC Whole net store in Moshi. Nine new whole IG2 nets coated with AIs, 100 mg/m^2^ alpha-cypermethrin and 200 mg/m^2^ chlorfenapyr, sourced from a community randomised trial in Tanzania [5], were assigned unique net identity codes 114B–117B and 128B–132B for the first four and next five respectively. Two untreated nets were assigned net identity codes 133B and 134B. First, the roof panels were separated from the side panels of untreated nets 133B and 134B; then similarly, roof panels were separated from side panels of IG2 nets 114B–117B and 128B–131B. This was done to prevent insecticide contamination and ensure uniform handling of treated and untreated panels of treatment and control panels during the rotations. Untreated net 133B was used as the negative control. From the other untreated net (134B), the roof and side panels were combined with the IG2 net panels, as illustrated in Fig. 1 and Additional file 1: Table S1, to form roof-treated IG2 and side-treated IG2.

Due to the zoophilic behaviour of the local *An. arabiensis* in the study site [25, 26], young cows were used as bait in this hut trial instead of human volunteers. The cows selected were 1- or 2-year-old non-lactating calves of similar size and weight which did not receive any insecticide treatment before or during the trial. No adult cows or bulls were used because they are too large to fit into the wooden containment enclosures (cow frames) and are more difficult to handle. Wooden containment enclosures of dimensions 140 cm width, 120 cm height and 180 cm length were made to hold cows within the huts. The total surface area covered with insecticide on the roof, side and whole IG2 nets was 2.88 m^2^, 12.24 m^2^ and 15.12 m^2^ respectively. Each net roof panel was stapled to a wooden detached roof panel of a cow frame and labelled with the net identity code shown in Additional file 1: Table S1. One line of stitching was cut from the side net panels that remained and the netting ‘rolled up’. The side netting was then put into a sealable plastic bag and labelled with the net identity. The trial treatments, which had either the roof-treated IG2 or side-treated IG2 panels only, were referred to as the “partially treated nets”.

All nets had 30 holes cut in the side panels to simulate the conditions of a torn net. Each hole (size 4 cm × 4 cm) was spaced evenly along the length of each side panel, with nine holes in two rows on each long side and six on each short side. This differs from the current WHO guidelines [27]; it is not unusual for nets to acquire this number of holes in use and remain functional [18, 28] (it is also more comfortable for the calf, which generates considerable heat). This also improves the power to detect a difference in blood feeding inhibition between the treatment arms of the trial and the control. Once all nets were prepared, they were transferred to the field station, ensuring that the IG2-treated netting did not come into contact with the untreated netting during transfer. Untreated roof netting fixed onto the detachable wooden roof panel of the cow frames was transported first to the field station and then followed by roof panels with IG2 netting. Side-treated IG2 and untreated net panels were transported in separate sealable plastic bags. All nets used were unwashed.

### Hut trial

Four experimental huts were used to compare four net treatments during the trial in a 4 × 4 Latin square design. To achieve not less than 80% power at an average mosquito density of 1 mosquito/hut/night, a total of four Latin square rotations were performed over 64 trial nights. This was sufficient to detect an effect size equivalent to the odds ratio of 0.23 in mosquito mortality in the roof, side and untreated trial arms compared to the whole IG2 net while considering random effects due to week, hut and overdispersion [29]. The net treatments were randomly allocated to huts and rotated every fifth day to a different hut. Cows were randomly rotated every day to a different hut. Cows, dressed in nappies to reduce hut and net contamination, were taken into the huts and put under the frames with net treatments each evening at 18:00 until mosquito collection the following morning at 06:00. Each morning two technicians collected mosquitoes from inside the rooms, nets, exit and verandah traps. Only the nets tested within each treatment arm of the trial were rotated daily during the next 4 days of the rotation, i.e. a different net from each treatment arm was used once in each 4-night rotation. In the partially treated net arms, either the roof-treated IG2 or the side-treated IG2 was rotated daily. As a precaution, in the side-treated IG2 arm, the untreated roof panel was removed first and stored outside the hut before the side panels were removed and replaced to prevent accidental contamination of the roof panels.

### Statistical analyses

The entomological efficacy of each treatment was compared to the untreated net in terms of mosquito immediate and delayed mortality, blood feeding inhibition, induced exiting and deterrence. Immediate and delayed mortality is the proportion of mosquitoes entering the huts that were found dead in the morning or after being caught alive and held for 72 h with access to glucose solution, with observations of mortality every 24 h. Blood feeding inhibition [27] is the proportional reduction of blood feeding in the treatment arms compared to the untreated net arm and was estimated using Eq. 1.

1 $$\mathrm{Blood}\, \mathrm{feed}\, \mathrm{inhibition} \left(\%\right)=\frac{\mathrm{Bu}-\mathrm{Bt}}{\mathrm{Bu}}\times 100$$

where Bu is the proportion of mosquitoes that are blood fed in the untreated arm and Bt is the proportion of mosquitoes that are blood fed in the treated arms.

Induced exiting is the percentage of mosquitoes collected from window and verandah traps in huts with treatments compared to those caught in the huts with untreated nets.

Deterrence [27] is the reduction in the number of mosquitoes caught in the huts with treated nets compared to huts with untreated nets and was estimated using Eq. 2.

2 $$\mathrm{Deterrence} \left(\%\right)=\frac{\mathrm{Du}-\mathrm{Dt}}{\mathrm{Du}}\times 100$$

where Du is the total number of mosquitoes that are caught in the huts with untreated nets and Dt is the total number of mosquitoes caught in the huts with treated nets.

The potential killing effect [27] of each treatment was estimated from Eq. 1.

3 $$\mathrm{Killing} \,\mathrm{effect} \left(\%\right)=\frac{\mathrm{Kt}-\mathrm{Ku}}{\mathrm{Tu}}\times 100$$

where Kt is the number of mosquitoes killed in the huts with treated nets, Ku is the number of mosquitoes killed in the huts with untreated nets, and Tu is the total number of mosquitoes collected from the huts with untreated nets.

Stata SE version 16.1 (StataCorp LCC, College Station, TX, USA) was used to process the experimental hut trial binary and count data into suitable formats to perform logistic and negative binomial regressions respectively. Grouped logistic regressions with a Logit link performed in Stata were used to analyse each outcome variable: mortality, blood feeding and exiting against trial arms with and without random effects due to variations in cows, huts, Latin square rotation of trial arms and trial night. For each outcome variable, grouped multiple logistic regressions were performed with untreated net, roof-treated IG2 and side-treated IG2 as references so that odds ratios (OR) could be compared between all trial arms. All graphs were created in Microsoft Excel Office 2019 (Microsoft Corporation, Redmond, WA, USA). The exact binomial (Clopper-Pearson) confidence intervals are reported on the graph. Negative binomial regression with a log link performed in Stata was used to measure the effect of trial arms on mosquito catches here referred to as deterrence while accounting for variation due to cows, huts, Latin square rotation and trial night. Predicted means from the negative binomial regressions are reported. Statistical significance was considered at α < 0.05.

## Results

A total of 367 female *An. arabiensis* were collected across all trial arms over 64 trap nights. Of these 113 (30.8%; 95% CI: 26.1–35.8%) were from the untreated net, 97 (26.4%; 95% CI: 22.0–31.2%) from the roof-treated IG2, 71 (19.3%; 95% CI: 15.4–23.7%) from the side-treated IG2 and 86 (23.4%; 95% CI: 19.2–28.1%) from the IG2 net trial arms.

### Mortality

**Fig. 2:**
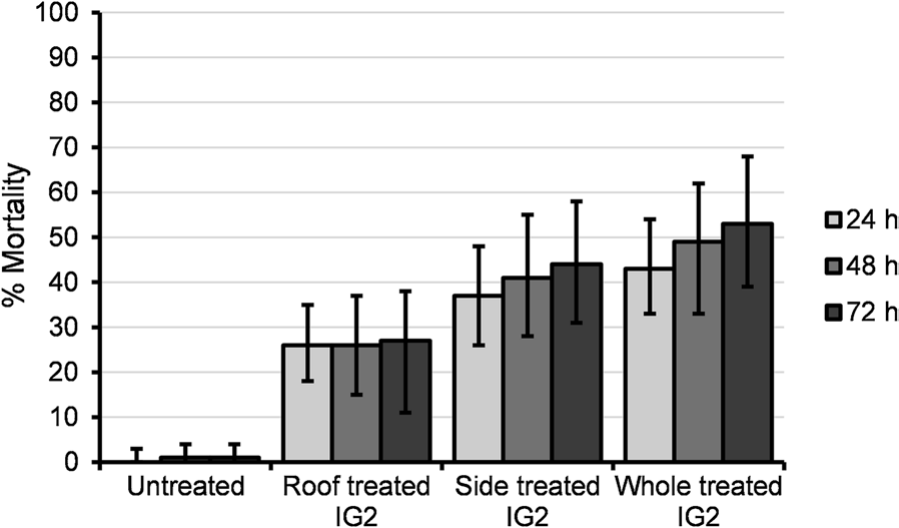
Mortality of wild free-flying *An. arabiensis* at 24, 48 and 72 h in trial arms. Error bars represent 95% confidence interval. Confidence intervals for 0% mortality are one sided

In total, 104 *An. arabiensis* were recorded dead with delayed mortality between 24, 48 and 72 h. At each time point, mortality was always higher in the IG2 net trial arm than the roof-treated IG2, side-treated IG2 and untreated net trial arms (Fig. 2). Overall, adjusted 72 h mortality was highest in the IG2, and it was significantly different from mortality in the roof-treated IG2 and untreated net (Table 1).

**Table 1:** Outcomes adjusted for random effects due to cows, huts, trial Latin square rotation and trial night

Outcome	Untreated	Roof-treated IG2	Side treated IG2	IG2
% Mortality at 72 h (95% CI)	1.2^a^ (0.0–3.9)	27.4^b^ (10.6–38.2)	44.4^bc^ (30.9–57.8)	53.3^c^ (39.1–67.5)
% killing effect (95% CI)	–	24.8 (17.1–33.8)	26.5 (18.7–35.7)	37.2 (28.3–46.8)
% Blood fed (95% CI)	21.9^a^ (9.6–34.2)	10.5^a^ (3.0–18.1)	13.7^a^ (4.3–23.0)	18.8^a^ (7.6–30.1)
% Blood feeding inhibition	–	52.0	37.4	14.1
% Exiting (95% CI)	92.5^a^ (82.5–100)	89.4^a^ (77.5–100)	97.1^a^ (91.2–100)	94.4^a^ (86.4–100)
Predicated mean catches (95% CI)	1.3^a^ (0.8–1.7)	1.7^a^ (1.1–2.4)	1.2^a^ (0.7–1.7)	1.4^a^ (0.9–2.0)

Treatments sharing the same letter script in each row do not differ significantly (*P* > 0.05, OR = 1 or rate ratio = 1)

Unadjusted and adjusted odds ratios for random effects due to cows, huts, trial Latin square rotation and night showed significant increases in mortality between treated IG2 (roof, side and whole) and untreated nets (Additional file 1: Table S2). There was a significant increase in the likelihood of mortality in the whole IG2 trial arm (*P* = 0.009, OR = 4.4, 95% CI = 1.4–13.3) compared to the roof IG2 trial arm after accounting for random effects due to cows, huts, Latin square rotation and trial night. There were no significant differences in mortality for both unadjusted and adjusted odds ratio between roof and side IG2 trial arms and side and whole IG2 trial arms (Additional file 1: Table S2). The killing effect was highest in the IG2 compared to roof-treated and side-treated IG2 trial arms (Table 1). The associated mortality per unit surface area treated with insecticide was 9 mosquitoes/m^2^ for the roof-treated IG2, 3 mosquitoes/m^2^ for sides-treated IG2 and 3 mosquitoes/m^2^ for IG2 net.

### Blood-feeding inhibition

Overall, 80/367 (21.8%) *An. arabiensis* took a blood meal, with the highest proportion being in the untreated net trial arm (Table 1). Of those that took a blood meal, 11 individuals died (two each in roof-treated and side-treated IG2, and seven in IG2). Four mosquitoes were caught in the net of which three were blood fed in the untreated arm and one unfed in the IG2 arm. Specifically, the associated percentages of blood meals were 21.9% in the untreated, 10.5% in the roof-treated IG2, 13.7% in the side-treated IG2 and 18.8% in the IG2 trial arms. There were no significant differences in blood feeding between the trial arms (Table 1). While the unadjusted odds ratio showed significant associations between blood feeding in the trial arms, upon accounting for random effects due to cow, hut, Latin square rotation and trial nights, the resulting adjusted odds ratio did not show any significant difference between blood feeding and trial arms (Additional file 1: Table S3).

### Hut exiting and deterrence

A total of 339/367 (92.4%) *An. arabiensis* were caught in the exit traps and verandahs. The tendency to exit was generally high across all the trial arms (Table 1) with percentage exiting of 92.5% in the untreated, 89.4% in the roof-treated IG2, 97.1% in the side-treated IG2 and 94.4% in the IG2 trial arms. After considering random effects, variation due to cows, huts, trial Latin square rotation and trial night, there was no significant treatment effects on exiting between roof-treated IG2, side-treated IG2, IG2 nets compared to the untreated net arm (Additional file 1: Table S4).

Deterrence for the roof-treated IG2, side-treated IG2 and IG2 net arms differed, being 14.2%, 37.2% and 23.9% respectively. However, a negative binomial regression of mosquito catches against treatments showed no significant deterrence in *An. arabiensis* entering roof-treated IG2, side-treated IG2 and IG2 huts compared to untreated huts while considering variation due to cows, huts, trial Latin square and trial night (Table 1 and Additional file 1: Table S5).

## Discussion

This study compared the efficacy of partially treated nets (roof-treated IG2, side-treated IG2), IG2 and untreated nets in hut studies. Evaluating the efficacy of partially treated nets is important as it may lead to new strategies for rational insecticide use and insecticide resistance management compared to whole-net treatments. Mortality and killing effects in both the partially treated and the IG2 nets were significantly higher than in untreated nets, an indication of their potential to reduce mosquito density and longevity, which could result in community protection if deployed in the majority of households [27]. Mortality in the IG2 net trial arm was significantly higher than in the roof-treated IG2 trial arm, suggesting that the whole IG2 net is more efficacious in killing *An. arabiensis* and would have a faster impact on mosquito density reduction if deployed than the roof-treated IG2 net. A previous study by Oxborough et al. [17] using roof, side and whole nets treated with a single pyrethroid lambda-cyhalothrin showed no significant difference in mortality among the three treatment arms. The difference in results between the present study and that of Oxborough et al. [17] might be due to differences in the properties of the insecticides used in the two studies. In the former trial, an irritant alpha-cyano pyrethroid was the sole AI, whereas in the current trial (done in the same location) a mixture of irritant pyrethroid and toxic pyrrole was used. The higher overall mortality observed in the current trial can be attributed to the toxic chlorfenapyr component of the mixture. Furthermore, the study by Oxborough et al. [17] used human sleepers while cows were used as attractants in the current study.

Unlike the former trial of Oxborough et al. [17] in experimental huts, there was greater mortality in the sides-only than in the roof-only IG2 netting arms, a confirmation that both the roof and side panels contributed to the higher mortality observed with IG2 nets. However, each panel's contribution to overall mosquito mortality requires further investigation as it could give insights into the development of LLIN based insecticide resistance management strategies. Though not statistically tested, the difference between 24 and 72 h mortality was highest in the whole IG2 nets (10 percentage points), followed by the side-only IG2 nets (seven percentage points) and then the roof-only IG2 nets (one percentage points). Of the two insecticides coated on IG2 nets, chlorfenapyr gives delayed mortality because it acts more slowly than the pyrethroid alphacypermethrin [12]; this could indicate that the total area of a partially treated net coated with chlorfenapyr influences *An. arabiensis* mortality. However, further investigations to determine the relative contribution of each insecticide on IG2 to mosquito mortality are required. This could give insights into whether insecticide properties such as excito-repellency could affect the efficacy of partially treated nets in terms of mortality.

Blood feeding was generally lower in the partially treated and whole IG2 nets than the untreated net. Even though the unadjusted odds ratio showed significant reductions in the likelihood of blood feeding in the partially and whole IG2 trial arms compared to untreated arm, these reductions were not significant after accounting for random effects due to cows, huts, Latin square rotation and trial night. This may suggest confounding factors which should be considered in similar or future studies. Among the factors that reduce the efficacy of pyrethroid nets to inhibit blood feeding is resistance to pyrethroids [30]. With pyrethroid resistance in *An. arabiensis* previously reported in the study area [23, 24], it could be responsible for the observed nonsignificant difference in blood feeding between the treated arms and the control. Further studies are required to investigate this possibility.

The nets used in this study had 30 holes cut in the side panels to reduce heat stress to the calf simulating a torn net instead of 6 holes recommended by WHO guidelines, simulating a damaged net. This limitation could influence the interpretation of our results especially on an outcome such as blood feeding, as increasing the number of holes in a net increases the proportion of mosquitoes that blood feed [31]. Therefore, the results should be interpreted with this number of holes in the nets used for this study. Several WHO Phase 3 studies of WHO approved LLIN record a mean of 20 holes after 2 years of use and ranging up to 30 after 3 years of use [18, 20].

Hut exiting adjusted for random effects due to cows, huts, Latin square rotation and trial night was generally high across the trial arms ranging from 89 to 97%, with 92% of *An. arabiensis* exiting in the untreated net arms. This observation is consistent with the exophilic nature of *An. arabiensis* reported in Lower Moshi, Tanzania [17, 25], and could have masked any induced exiting effect due to the pyrethroid component in partially treated and IG2 nets. Furthermore, the partially treated and IG2 nets showed a deterrence effect based on a calculation that used the total number of mosquitoes caught in the trial arm. However, statistical analysis of the difference between the adjusted predicated mean mosquito catches between the trial arms indicated that the difference in deterrence between the partially treated and IG2 nets was not statistically significant.

The results of this study are consistent with the findings of others who have conducted research on mosaic or 2-in-1 partially treated net [15, 17] and showed maximal killing could be achieved through the spatially restrictive application of a more toxic or more expensive partner insecticide. This does not apply to IG2, which is affordable and, as shown here, is best used as a five-panel treated net.

Finally, results from the recently published study conducted by Sutcliffe and Yin [16] suggest that the direction of air movement within huts may influence where on a net mosquito will make first contact upon entering a hut. Air velocity measurements inside huts were not included in this study, but consideration of this should be made when designing future studies. Previous studies have reported that *An. arabiensis* seeking a blood meal make multiple brief contacts on the roof compared with side panels of human occupied LLINs [4]. In this study, cows were used instead of humans, which could have implications on generalising the results across hosts. However, the observed higher killing efficiency in the roof-treated IG2 arm (9 mosquitoes/m^2^, i.e. 3 times as many were killed by the treated roof/m^2^ than by the treated sides/m^2^), despite having the smallest surface area covered with insecticide netting, suggests that *An. arabiensis* also makes multiple contacts on a roof compared with the side panel of cow occupied LLIN. Nevertheless, the possible effect on outcomes due to the host used as attractant should be further investigated. This observation could also be an indication that future LLIN designs with more than one AI should consider spatially restricting non-excito-repellent insecticides to the roof panel.

## Conclusion

This study found the induced mortality of the partially treated net compared to the untreated net increased in the order roof only, side only and whole IG2 net. In addition, the whole IG2 net was more efficacious than the roof only IG2 net in terms of mortality and killing effect. Therefore, under the specific conditions of this study, using an East African hut design with cows as bait and a field strain of *An. arabiensis*, a roof only treatment is not appropriate to use as an LLIN design to reduce the insecticide loading with an IG2 net.

## Supplementary Information

**Additional file 1: Table S1**. Experimental hut trial arms.** Table S2**. Measures of association between treatment arms and mortality.** Table S3**. Measures of association between treatment arms and blood feeding.** Table S4**. Measures of association between trial arms and exiting of huts.** Table S5**. Measures of effect between trial arms and hut entry (deterrence).

## Data Availability

The dataset generated and analysed during this study is available from the corresponding author upon reasonable request.

## Abbreviations

AI: Active ingredient
IG2: Interceptor® G2 net
KCMUCo-PAMVERC: Kilimanjaro Christian Medical University College-Pan African Malaria Vector Research Consortium
LLIN: Long-lasting insecticidal net
