# Supplementary material for: Efficacy of bednets with dual insecticide-treated netting (Interceptor® G2) on side and roof panels against Anopheles arabiensis in north-eastern Tanzania
Source: Parasit Vectors. 2022 Sep 15;15:326. doi: 10.1186/s13071-022-05454-w (PMC9479251; doi:10.1186/s13071-022-05454-w)
Supplement: Supplementary file 1 — Additional file 1: Table S1. Experimental hut trial arms. Table S2. Measures of association between treatment arms and mortality. Table S3. Measures of association between treatment arms and blood feeding. Table S4. Measures of association between trial arms and exiting of huts. Table S5. Measures of effect between trial arms and hut entry (deterrence). [file 13071_2022_5454_MOESM1_ESM.docx]

**List of Supplementary Tables**

**Table S1: Experimental hut trial arms**

| **Roof panel** | **Side panels** | **Net ID codes** |
| --- | --- | --- |
| IG2 | Untreated (BO XIN net) | 128B-roof, 129B-roof, 130B-roof, 131B-roof |
| Untreated (BO XIN net) | IG2 | 128B-side, 129B-side, 130B-side, 131B-side |
| IG2 | IG2 | 114B, 115B, 116B, 117B |
| Untreated (BO XIN net) | Untreated (BO XIN net) | 133B |

**Table S2: Measures of association between treatment arms and mortality**

| **Reference** | **Exposure** | **UOR (95% CI)** | **P-value, Z-value** | **AOR (95% CI)** | **P-value** |
| --- | --- | --- | --- | --- | --- |
| Untreated | Roof | 47.76 (6.36 – 358.66) | <0.001* | 51.00 (4.76 – 546.21) | 0.001* |
| Untreated | Side | 86.80 (11.47 – 656.81) | <0.001* | 137.6 (12.2 – 1553.2) | <0.001* |
| Untreated | Whole | 112.00 (14.95 – 838.87) | <0.001* | 222.95 (19.07 – 2606.01) | <0.001* |
| Roof | Side | 1.81 (0.96 – 3.44) | 0.067 | 2.69 (0.93 – 7.74) | 0.065 |
| Roof | Whole | 2.34 (1.28 – 4.29) | 0.006* | 4.37 (1.44 – 13.26) | 0.009* |
| Side | Whole | 1.29 (0.69 – 2.43) | 0.429 | 1.62 (0.56 – 4.71) | 0.375 |

UOR is unadjusted odds ratio; AOR is adjusted odds ratio for random effects; CI is confidence interval; *** Statistically significant.

**Table S3: Measures of association between treatment arms and blood feeding**

| **Reference** | **Exposure** | **UOR (95% CI)** | **P-value, Z-value** | **AOR (95% CI)** | **P-value, Z-value** |
| --- | --- | --- | --- | --- | --- |
| Untreated | Roof | 0.20 (0.09 – 0.43) | <0.001* | 0.32 (0.08 – 1.28) | 0.107 |
| Untreated | Side | 0.29 (0.13 – 0.62) | 0.002* | 0.47 (0.12 – 1.88) | 0.282 |
| Untreated | Whole | 0.50 (0.26 – 0.94) | 0.032* | 0.77 (0.20 – 3.02) | 0.709 |
| Roof | Side | 1.43 (0.56 – 3.63) | 0.457 | 1.47 (0.36 – 5.95) | 0.592 |
| Roof | Whole | 2.48 (1.08 – 5.65) | 0.033* | 2.43 (0.61 – 9.67) | 0.207 |
| Side | Whole | 1.73 (0.75 – 4.01) | 0.201 | 1.66 (0.41 – 6.77) | 0.481 |

UOR is unadjusted odds ratio; AOR is adjusted odds ratio for random effects; CI is confidence interval; *Statistically significant.**Table S4: Measures of association between trial arms and exiting of huts**

| **Reference-** | **Exposure** | **UOR (95% CI)** | **P-value, Z-value** | **AOR (95% CI)** | **P-value, Z-value** |
| --- | --- | --- | --- | --- | --- |
| Untreated | Roof | 0.69 (0.28 – 1.67) | 0.408 | 0.47 (0.02 – 11.19) | 0.638 |
| Untreated | Side | 6.80 (0.85 – 54.28) | 0.071 | 6.32 (0.13 – 301.54) | 0.350 |
| Untreated | Whole | 1.57 (0.52 – 4.78) | 0.425 | 1.83 (0.10 – 34.06) | 0.683 |
| Roof | Side | 9.88 (1.25 – 77.88) | 0.030* | 13.57 (0.61 – 297.35) | 0.098 |
| Roof | Whole | 2.29 (0.77 – 6.78) | 0.136 | 3.94 (0.15 – 106.13) | 0.414 |
| Side | Whole | 0.23 (0.03 – 2.03) | 0.186 | 0.29 (0.01 – 11.11) | 0.506 |

UOR is unadjusted odds ratio; AOR is adjusted odds ratio for random effects; CI is confidence interval; ***Statistically significant.**Table S5: Measures of effect between trial arms and hut entry (deterrence)**

| **Trial arm** | **Rate ratio^1^ (95% CI)** | **P-value** |
| --- | --- | --- |
| Untreated | 1 | - |
| Roof-treated IG2 | 1.37 (0.81 – 2.32) | 0.243 |
| Side-treated IG2 | 0.94 (0.55 – 1.61) | 0.828 |
| IG2 | 1.12 (0.66 – 1.90) | 0.677 |

CI is confidence interval. ^1^ Rate ratio is a measure of effect that mosquitoes chose other treatments instead of the control.
